# Multi‐planner validation of RapidPlan knowledge‐based model for volumetric modulated arc therapy in prostate cancer

**DOI:** 10.1002/acm2.14223

**Published:** 2023-11-27

**Authors:** Isra Israngkul Na Ayuthaya, Taweap Sanghangthum, Puntiwa Oonsiri, Sornjarod Oonsiri, Mintra Keawsamur, Sirinya Ruangchan, Chulee Vannavijit, Jaruek Kanphet, Sakda Kingkaew, Mananchaya Vimolnoch, Tanawat Tawonwong, Nuttha Plangpleng

**Affiliations:** ^1^ Division of Radiation Oncology Department of Radiology King Chulalongkorn Memorial Hospital Bangkok Thailand; ^2^ Division of Radiation Oncology Department of Radiology Faculty of Medicine Chulalongkorn University Bangkok Thailand

**Keywords:** knowledge‐based planning, prostate cancer, RapidPlan model, treatment planning, VMAT

## Abstract

**Purpose:**

To investigate the performance of a model‐based optimization process for volumetric modulated arc therapy (VMAT) applied to prostate cancer patients with the multi‐planner.

**Methods and Materials:**

The 120 prostate plans for VMAT treatment were entered into the database system of the RapidPlan (RP) knowledge‐based treatment planning. The treatment planning data for each plan was used to create and train the RP model. Twelve prostate cancer cases were selected and were used for planning by a manual of 12 planners based on the clinical protocol for dose constraints. Then, the treatment plans for each patient were compared with the RP model plans and analyzed with Wilcoxon tests.

**Results:**

On average, the RP models can estimate comparable doses among all planner plans and clinical plans for the PTV, which D_max_, D_95%_, D_98%_, HI, and CI were used to evaluate. For the normal organ doses of the bladder, rectum, penile bulb, and femoral head, all RP model plans showed comparable or better dose sparing than all planner plans and clinical plans. Moreover, the average planning time of the RP model was faster than manual plans by about two times. The RP model can significantly reduce the variation dose of the normal organs compared with the manual plans among the planners.

**Conclusion:**

The automated plans of the RP model might benefit from further fine‐tuning of the dose constraints of the normal organs, although both procedure plans are acceptable and fulfill the clinical protocol goals so that the RP model can enhance the efficacy and quality of plans.

## 1 INTRODUCTION

Intensity‐modulated radiation therapy (IMRT) and volumetric‐modulated arc therapy (VMAT) are commonly used to treat prostate cancer patients in clinical treatments. They are able to deliver the maximum dose to the target and the minimum dose to the normal organs, which is the goal of radiotherapy. Treatment results show good response in patients with early‐stage prostate cancer^1^ and can minimize the dose to the organ at risk. IMRT and VMAT plans use the inverse planning technique to optimize and calculate the dose for the patient. A trial‐and‐error approach is used for the optimization process, which completely consumes the time for a calculated plan. Each treatment plan is highly tailored to the individual patient. It is important to understand the sources of variability in the system that could affect the overall quality of the treatment plan's output. Although the final plans receive the dose within the dose criteria, the dose variation between each planner is still wide.^2^ This is an effect of the quality of the plan. The planners must improve and decrease the variation in any treatment plans, which knowledge‐based (KB) treatment planning can help minimize.^3^, ^4^

The RapidPlan (RP) model is a KB treatment planning system that uses machine learning to improve the efficiency of the plan released commercially by Varian Medical Systems. The RP model is a new feature of the Eclipse treatment planning system that requires the anatomical and dosimetric data of the patients and correlates them via training and learning in the model. The model needs a large number of patients to create the best model for use. Then, the model can be used to create a suitable estimate of the achievable dose distribution for prospective cases and the optimization constraints through automation. It can help the planner improve plan quality by reducing the variation in dosimetry of target and normal organs which has been investigated in many studies.^5^, ^6^, ^7^, ^8^ Some general trends were observed in these studies, including generally improved plan quality, decreased inter‐clinician variability, and the transfer of planning expertise from more experienced to less experienced planners.^9^ Moreover, Israngkul‐Na‐Ayuthaya I et al.^10^ also reported that the RP model can reduce planning time. Although the manufacturer suggests utilizing at least 20 plans for learning in the RP model, the appropriate number of plans should be as large as possible for high‐performance model. From the basics of statistical principles, the size of the sample is very important for getting accurate, statistically significant results and running the study successfully, which agrees with the result of this study.^10^ The results showed a better dose in some OARs for a large number of the plans used for the RP model. Thus, this study aims to investigate the performance of a model‐based optimization process for volumetric modulated arc therapy (VMAT) applied to prostate cancer patients with the RP model.

## 2 METHODS AND MATERIALS

### 2.1 Knowledge‐based learning library

The VMAT plans of 120 prostate cancer patients, which were previously manually planned to use the Eclipse treatment planning system (Varian Medical Systems, Palo Alto, CA) and delivered in clinical treatment, were randomly collected from the database between 2017 and 2020. The selected patients had localized prostate cancer that had not yet spread outside the prostate. The patients with T1‐T2c prostate cancer were used to train for the RP planning model. All cases were anonymized before being collected in the database. The data information for each plan consisted of the CT image, normal organ volumes, target volumes, and dose distributions. All treatment plans were performed using the VMAT technique using 6 MV photon beams with two full rotation arcs. The prescribed dose was 79.2 Gy in 44 fractions as the planning target volume (PTV). The optimization and calculation process used the dose constraint of PTV and organs at risk (OARs) as the bladder, rectum, penile bulb, and femoral head according to the RTOG 0126 randomized clinical trial recommendation.^10^ Then, the objective organs were analyzed for the DVH constraint definition in Table 1.

**TABLE 1:** Dose constraints for dose optimization and calculation.

Organs	Dose constraints
PTV	D_max_ ≤ 107%, D_95%_ ≥ 95%, and D_98%_ ≥ 93%
Bladder	V_80Gy_ ≤ 15%, V_75Gy_ ≤ 25%, and V_65Gy_ ≤ 50%
Rectum	V_75Gy_ ≤ 15%, V_70Gy_ ≤ 25%, V_65Gy_ ≤ 35%, and V_60Gy_ ≤ 50%
Penile bulb	V_50Gy_ ≤ 10% and D_mean_ ≤ 48 Gy
Femoral heads	D_mean_ ≤ 52.5 Gy

### 2.2 RapidPlan model configuration

Initially, the geometric and dosimetric parameters of each plan were imported and trained in a RP model configuration.^12^ The minimum number of training cases required for the RP model was 20 cases. In this study, the model configuration was based on the number of 120 prostate VMAT plans for import and training into the RP model system, which was the appropriate number of plans in the database of RP model investigated in the previous study.^10^

After import and training, the RP model was able to generate dose constraints for automatic optimization that fulfilled the clinical acceptability requirements shown in Table 1. In the optimization process of Eclipse treatment planning, the priority values determined the important level of each organ, and the PTV set the important organ to high values. In the optimization process, the treatment planning computer calculated the plans until they were completed through automation. The RP model was optimized, and the doses were calculated in the new treatment plans.

### 2.3 Generating new treatment plans

After the RP model was finished with the configuration process, 12 new prostate cancer cases were selected for comparison of the dose between the RP model and the manual planner calculation. They needed to be treated completely in clinical treatment, but their plans had never been used to collect data in an RP model. They were used to investigate and compare the different doses of planning target volume (PTV) and organs at risk (OARs). In each plan, the VMAT technique with two full arcs and a 6 MV photon beam was used to plan. The dose prescription of 79.2 Gy in 44 fractions was applied to PTV. For the RP model, the dose objectives were generated automatically for PTV and OARs in the optimization and calculation of dose procedures. The dose‐volume histograms (DVHs) of PTV and OARs were analyzed and compared to the dose criteria in Table 1. The final dose must meet the criteria before being accepted for the plan to be analyzed for the next step. The results of the RP model were analyzed and compared with the manual plans from 12 medical physicists and clinical plans for each patient. The clinical plans were the manual plans from treatment planners that were used to treat the patient in clinical treatment.

Twelve planners were divided into three groups by their working experience: more than 10 years (G1), 5–10 years (G2), and less than 5 years (G3). The number of planners in each planning group was four. All planners created and optimized all plans by manual but fulfilled the clinical acceptability requirements in Table 1, and the planning time must be completed in 3 weeks. This method can control the reasonable variation and quality of plans between multiple different planners. The planners must generate their best plan through acceptable and fulfilling the clinical protocol goals and submit their results for analysis and comparison with the RP plan model. The PTV must receive the prescribed dose, and the OARs must receive a dose as low as possible. Radiation oncologists evaluated and approved all the plans as DVHs and dose distribution reviews. They can be utilized to treat patients in clinical settings.

### 2.4 Dose comparison between each RP model and manually optimized plans

Each plan used the VMAT technique with two fully rotating 6 MV photon beams for dose optimization and calculation. In the optimization process, the dose objectives and optimization priorities were set manually, the same as in clinical treatment, for each planner, while they were set automatically for the RP model. The doses of all plans must pass the dose limit of the dose constraints shown in Table 1 and physician reviews to be accepted. Then, the doses of each planner were compared with the RP model. Each dose constraint for PTV and OARs was reported from the DVH lines.

The RP plans and manual plans were compared using the Wilcoxon signed‐rank test, and a difference of *p*‐value < 0.05 was deemed significant.

For the PTV, the conformity index (CI) was used to evaluate the quality of the treatment plan and was calculated by Equation 1.^13^, ^14^, ^15^

(1) $$CI\;=\;\frac{PTV_{P}}{TV}$$

where PTV_P_ is the volume of the target receiving the prescription dose and TV is the target volume. The conformity of the PTV dose distribution is represented by the CI value, which indicates that the perfect dose conformity is unique.

The homogeneity index (HI) of the PTV is defined as Equation 2.^16^

(2) $$HI\;=\;\frac{D_{2\%}-D_{98\%}}{D_{p}}$$

where D_2%_ is the minimum dose to 2% of the target volume, indicating the maximum dose; D_98%_ is the minimum dose to 98% of the target volume, indicating the minimum dose; and D_p_ is the prescribed dose. The ideal dose homogenous of PTV is represented by the HI value of null.

## 3 RESULTS

The average dose results from 12 planners, clinical plans and the RP model calculated in 12 prostate patient plans are shown in Table 2. In RP model plans and all manual plans, the average PTV D_95%_ was a comparable dose (79.25 ± 0.01 Gy, *p* > 0.05). The average PTV D_98%_ of all manual plans was slightly higher than RP model with no statistical difference (RP: 77.92 ± 0.16 Gy, all planners: 78.08 ± 0.25 Gy, and clinical plans: 78.04 ± 0.22 Gy, *p* > 0.05). The average PTV D_2%_ was RP model: 83.53 ± 0.32 Gy, all planners: 83.27 ± 0.45 Gy, and clinical plans: 83.84 ± 0.52 Gy. For PTV D_2%_, the average dose of all planners was lower than the RP model (*p* < 0.05). On the other hand, the average dose of clinical plans was higher than the RP model (*p* < 0.05). However, all plans were acceptable for clinical use for treating the patient because dose constraints and distributions were satisfied.

**TABLE 2:** Average dose comparison of the PTV from 12 new prostate cancer VMAT plans in each dose parameter between RP model, 12 planners and clinical plans (CL).

Organ	Dose parameters	Model	Mean (Gy) ± SD	*p*‐value
PTV	D_95%_	RP	79.25 ± 0.01	Ref
		ALL Planners	79.25 ± 0.01	0.655
		CL	79.25 ± 0.01	0.677
	D_98%_	RP	77.92 ± 0.16	Ref
		ALL Planners	78.08 ± 0.25	0.058
		CL	78.04 ± 0.22	0.051
	D_2%_	RP	83.53 ± 0.32	Ref
		ALL Planners	83.27 ± 0.45	0.034
		CL	83.84 ± 0.52	0.029

For homogeneity index and conformity index, the average HI of each planning plan was in the range of 0.06–0.07. As shown in Table 3, the average CI of all planning plans was 0.96. HI and CI were not different between RP model and all manual plans.

**TABLE 3:** The CI and HI values of PTV for RP model (RP), all planners (ALL), each planner group (G1, G2, and G3), and clinical plans (CL).

Parameters	Model	Mean
CI	RP	0.96
	ALL	0.96
	G1	0.96
	G2	0.96
	G3	0.96
	CL	0.96
HI	RP	0.07
	ALL	0.06
	G1	0.06
	G2	0.06
	G3	0.06
	CL	0.06

For the rectum, the average dose volumes of 12 patient plans for V_75Gy_, V_70Gy_, V_65Gy_, and V_60Gy_ are shown in Table 4, which was a readout from the DVH. The results from both manual plans from all planners and automatic RP plans passed all criteria. The average rectum doses in the RP model were lower than all planner groups and clinical plans at V_75Gy_, V_70Gy_, V_65Gy_, and V_60Gy_ by *p* < 0.05. None of the plans generated a rectum dose over the limit of the dose constraints.

**TABLE 4:** Average rectum dose from 12 prostate cancer VMAT plans for RP model (RP), all planners (ALL), each planner group (G1, G2, and G3), and clinical plans (CL).

Organ	Dose constraints	Model	Mean ± SD (%)	*p*‐value
Rectum	V_75Gy_ < 15%	RP	11.41 ± 2.1	Ref
		ALL	12.33 ± 1.5	0.034
		G1	12.05 ± 1.7	0.049
		G2	12.32 ± 1.4	0.034
		G3	12.44 ± 1.6	0.028
		CL	12.06 ± 2.3	0.042
	V_70Gy_ < 25%	RP	15.03 ± 2.5	Ref
		ALL	16.63 ± 1.8	0.008
		G1	16.41 ± 2.1	0.015
		G2	16.82 ± 1.7	0.004
		G3	16.53 ± 1.9	0.028
		CL	16.16 ± 2.8	0.039
	V_65Gy_ < 35%	RP	18.21 ± 2.4	Ref
		ALL	20.44 ± 2.1	0.006
		G1	20.22 ± 2.5	0.008
		G2	20.24 ± 2.3	0.004
		G3	20.81 ± 2.1	0.019
		CL	19.99 ± 2.5	0.005
	V_60Gy_ < 50%	RP	21.32 ± 3.8	Ref
		ALL	24.25 ± 3.3	0.005
		G1	23.81 ± 2.8	0.005
		G2	24.84 ± 2.7	0.005
		G3	24.13 ± 3.1	0.012
		CL	23.96 ± 2.9	0.005

For the bladder, the comparison of the dose volume parameters for the bladder between the RP model and all manual plans is shown in Table 5. For the V_80Gy_, the average dose volume of the RP model was comparable to or slightly lower than all planners by *p* > 0.05. While comparison values with each group of planners were higher than G1 and clinical plans, they were lower than G2 and G3 (*p* > 0.05). For the V_75Gy_ and V_65Gy_, the average dose volume of the RP model was lower than all planners, each group of planners, and the clinical plan (*p* < 0.05), except the average dose volume of the V_75Gy_ was lower than G1 and clinical by *p* > 0.05. All plans can be optimized and calculated to achieve the doses within the dose objectives of the bladder.

**TABLE 5:** Average bladder dose from 12 prostate cancer VMAT plans for RP model (RP), all planners (ALL), and each planner group (G1, G2, and G3).

Organ	Dose constraints	Model	Mean ± SD (%)	*p*‐value
Bladder	V_80Gy_ < 15 %	RP	5.66 ± 2.7	Ref
		ALL	5.68 ± 2.8	0.722
		G1	5.59 ± 2.7	0.289
		G2	5.81 ± 2.8	0.077
		G3	5.64 ± 2.8	0.583
		CL	5.61 ± 2.8	0.591
	V_75Gy_ < 25%	RP	7.79 ± 3.7	Ref
		ALL	8.30 ± 3.8	0.016
		G1	8.13 ± 3.7	0.059
		G2	8.43 ± 3.8	0.008
		G3	8.32 ± 3.8	0.010
		CL	8.15 ± 3.7	0.052
	V_65Gy_ < 50%	RP	10.47 ± 4.5	Ref
		ALL	11.26 ± 4.8	0.004
		G1	10.98 ± 4.7	0.041
		G2	11.48 ± 4.9	0.005
		G3	11.52 ± 4.8	0.002
		CL	11.17 ± 4.8	0.021

For the femoral heads, the average mean doses are shown in Table 6. The mean dose calculated by the RP model was less than those calculated by all manual plans (*p* < 0.05 for all). The RP plans were not only decreasing the femoral head doses but also reducing the variation that supported by SD value.

**TABLE 6:** Average femoral heads dose from 12 prostate cancer VMAT plans for RP model (RP), all planners (ALL), and each planner group (G1, G2, and G3).

Organ	Dose constraints	Model	Mean (Gy) ± SD	*p*‐value
Femoral Heads	D_mean_ < 52.5 Gy	RP	14.92 ± 1.5	Ref
		ALL	16.36 ± 2.1	0.004
		G1	15.97 ± 2.0	0.019
		G2	16.67 ± 2.0	0.003
		G3	16.45 ± 2.3	0.010
		CL	16.15 ± 1.9	0.012

Table 7 shows comparison of the average mean dose of penile bulb for the RP plan, all planners, and clinical plans. For the average mean dose, RP model was lower than all planners and clinical plans by *p* < 0.05.

**TABLE 7:** Average penile bulb dose from 12 prostate cancer VMAT plans for RP model (RP), all planners (ALL), and each planner group (G1, G2, and G3).

Organ	Dose constraints	Model	Mean ± SD (Gy)	*p*‐value
Penile Bulb	D_mean_ < 48 Gy	RP	15.61 ± 9.7	Ref
		ALL	20.19 ± 11.5	0.004
		G1	17.85 ± 9.5	0.019
		G2	22.48 ± 12.9	0.003
		G3	20.23 ± 11.6	0.010
		CL	18.31 ± 10.5	0.015

**FIGURE 1:**
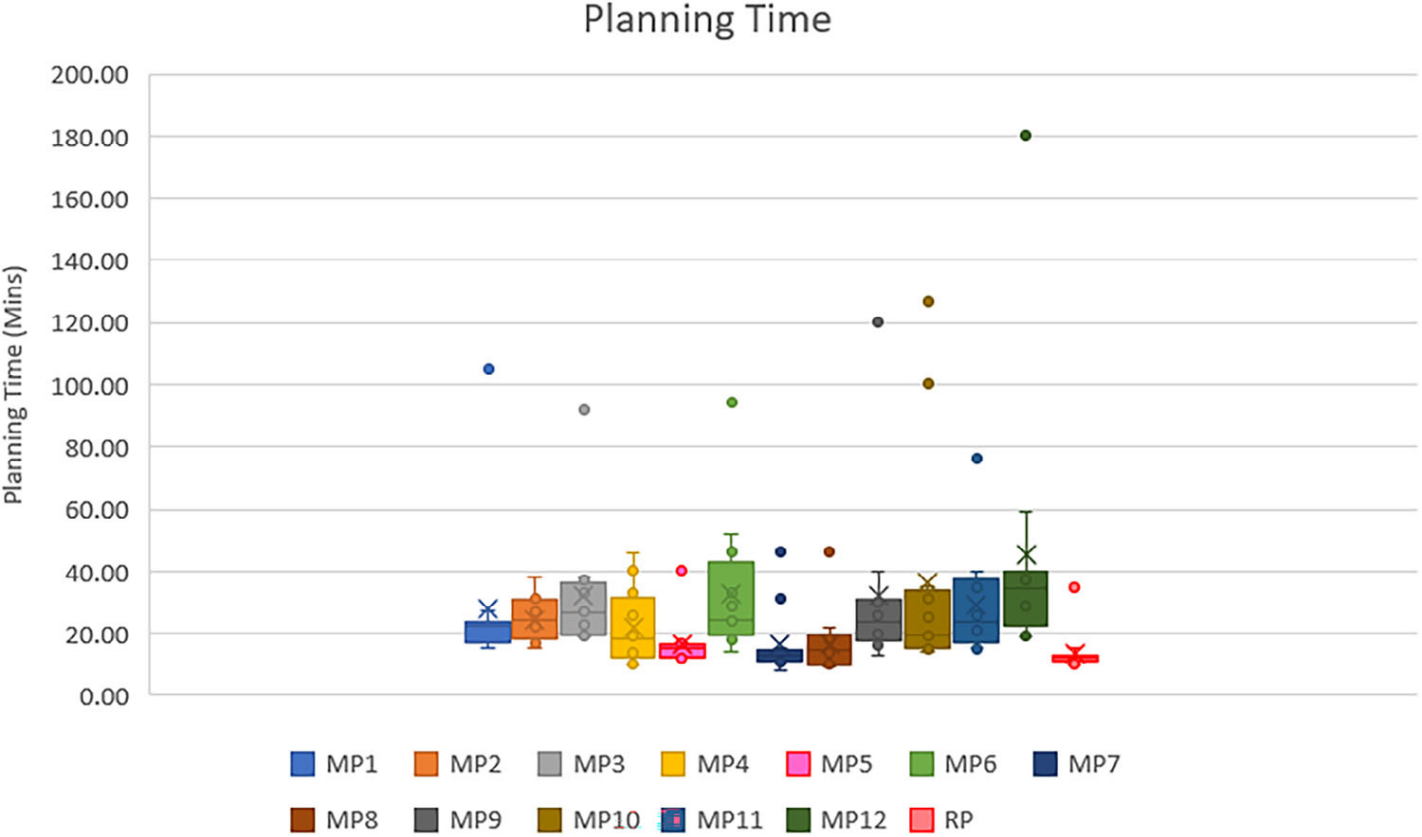
Planning time of RP model and each planner (MP1‐12) in 12 cases of prostate cancer.

**FIGURE 2:**
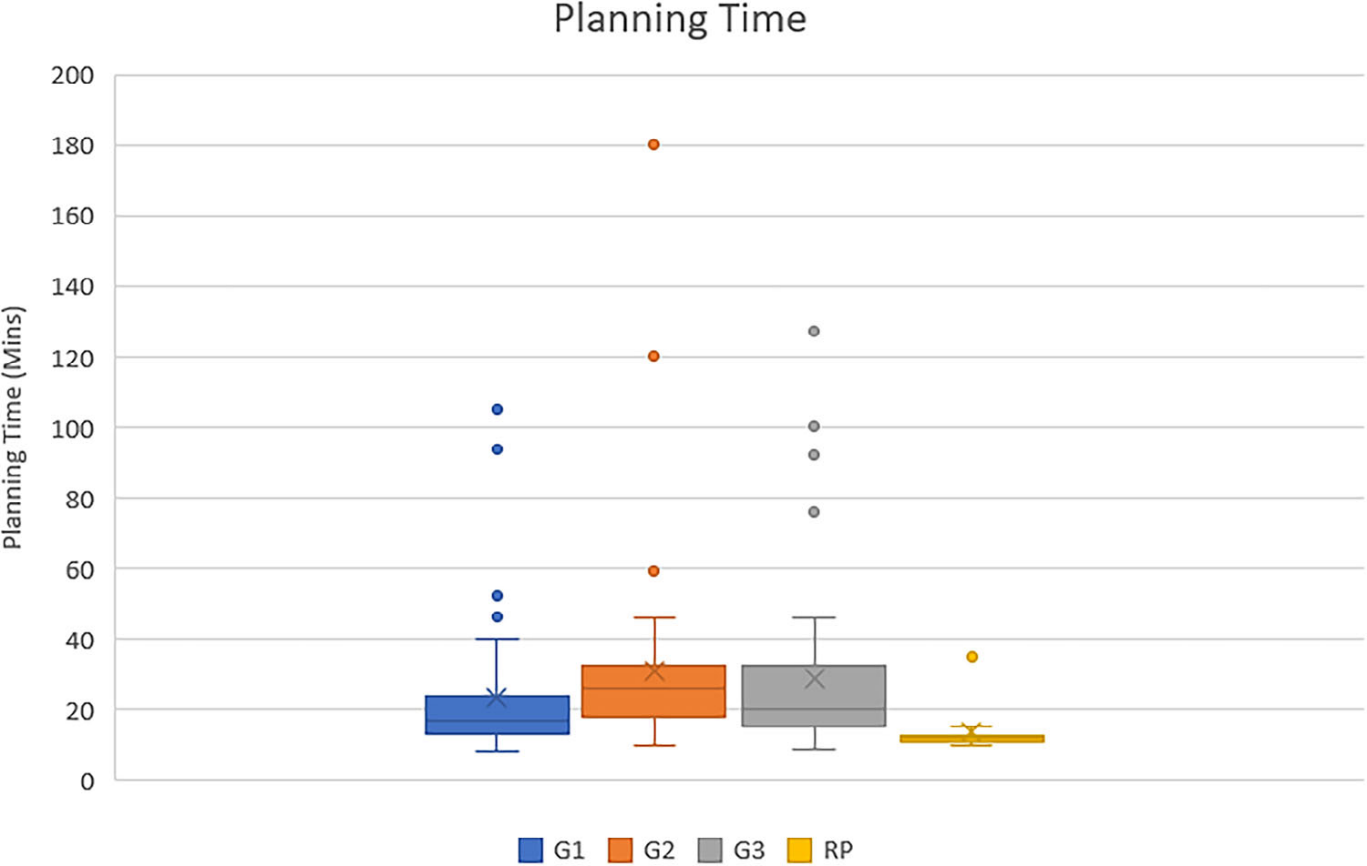
Planning time of RP model and each planner group (G1‐G3).

The average planning time of the RP model and all planners was 13.67 ± 6.56 min and 27.55 ± 23.74 min. The RP model can reduce the planning time by about two times and present very small variation times when compared to the manual plan in the prostate cancer plans. The variation in planning time between the RP model and each planner is shown in Figure 1. For each planner group, the planning time of each group differs significantly with the RP model by *p* < 0.05 (G1: 23.33 ± 18.82 min, G2: 30.81 ± 27.89 min, G3: 28.50 ± 23.77 min, RP: 13.67 ± 3.78 min). During the comparison between each planner group, the planning time of G1 and the other groups showed a significant difference of *p* < 0.05. On the other hand, the planning times of G2 and G3 showed no significant difference (*p* = 0.22). The Wilcoxon signed rank test was used to test the statistically significant difference. The variation in planning time between the RP model and each planner is shown in Figure 2.

## 4 DISCUSSION

This study investigated the performance of knowledge‐based RP treatment planning for VMAT prostate cancer patients in comparison with the manual plan by a multi‐planner. The 120 prostate plans were used for RP training. The 12 more prostate cancer cases were planned by 12 various experienced physicists and compared with the RP model. The VMAT plan needs to be optimized through the inverse planning procedure. The experience of planners is essential for the optimization of a trial‐and‐error approach. VMAT planning takes a long time in the optimization and calculation process, depending on the complexity of the plan and the planner's experience. RP knowledge‐based planning, which uses automated inverse planning procedures, can reduce planning time and variation in plan quality, as previously reported.^7^, ^17^ Before using the RP model for clinical treatment, its performance should be verified and compared to that of a manually optimized plan or a completely clinical treatment plan. A model can be confidently applied to the clinic to increase the efficiency of the plan compared to the dose with multi‐planners with different experiences in planning prostate cancer.

For the optimization process, each plan was set as the first‐order priority for PTV in the optimization table, and to control 95% of the PTV dose, the patient must receive the prescribed dose. So, the doses of PTV D_95%_ are not significantly different compared between the RP model and the manual plan by the multi‐planner, and manually clinical plans. All final plans achieve the dose constraints for PTV and the same quality of plan conformity and homogeneity. The RP model has a slightly higher dose of D_max_ for PTV. This issue can be improved by re‐arranging the priority value in the optimization process or re‐manualizing optimization after reducing the hot spot. Kubo et al.^17^ reported the comparison of the dose calculation from the RP plan and the clinical manual plan, in which their results showed similar values of D_95%_, D_2%_, HI, and CN. On the other hand, Hussein et al.^7^ reported that the RP model can exhibit slightly better conformity to PTV than the original manual optimization plans.

For normal organs, the RP model had lesser normal organ doses than each manual plan, both all planners and clinical plans, for all dose parameters; however, there was a significant difference except for the bladder dose of V_80Gy_ ≤ 15%, where the dose differences were not statistically significant. The RP model helps the planner reduce the dose for every dose constraint. When focusing on planner groups, some dose constraints of the normal organs had no significant difference between the RP model and the planner group of G1 as the expert with the longest planning experience, such as the bladder dose and penile bulb. The comparison of the dose between the RP model and the planner groups of G2 (intermediate experience planner) and G3 (beginner planner) showed a significant difference in dose for some organs. There was a statistically significant dose difference except for the bladder V_80Gy_ < 15%. The RP model helps the planner plan a suitable dose independent of experience, but the model must be trained and learned on suitable data‐based and learning cases. If the number of patient plans for training and learning increases on a data‐based model, the efficiency and performance of the RP model can be enhanced, especially in bladder and rectum dose reduction. It can reduce and spare the dose of normal organs as low as possible in each plan. The suggestion of a vendor is 20 cases at least for training in prostate cancer plans; however, 60−120 training cases are recommended in the RP model to have high‐quality VMAT plans based on our experience and research.^7^, ^10^, ^17^ RP model must be verified, and the performance based on each institute investigated before using the clinical plans. The suitable procedure to learn and train the data‐based and setup optimization parameters ensures the best efficiency of the plans. Sometimes, the RP plan is not the perfect tool. It must be used manually during the optimization procedure to improve and control the organ doses until they are received under the dose constraints, especially the normal organs with PTV overlap.^10^, ^17^ The number of plans for training in the RP system led to a higher quality of conformity, coverage, and efficiency compared to clinical plans.^7^

For the planning time, the RP model can reduce the planning time by about two times when compared to manual plans, according to statistical significance. Inter‐planner treatment planning time showed different significance between the senior group (G1) and the junior groups (G2 and G3), while there was no difference between the G2 and G3 groups. Because of the heuristics used by planners, there is still inter‐planner variation in the plan quality, particularly for complex VMAT plans.^18^

## 5 CONCLUSIONS

The Varian RapidPlan knowledge‐based planning can produce comparable or better doses with the clinical manual in a single optimization for prostate cancer cases. The RP model can enhance the efficacy and quality of plans; furthermore, it helps planners reduce the time consumed per plan. Finally, RapidPlan during the optimization of treatments for prostate cancer induces a significant increase in plan quality and a contextual reduction in plan variability. RapidPlan is proven to be a valuable tool to leverage to ensure better treatment plan quality and enhance the consistency of plan quality.

## AUTHOR CONTRIBUTIONS

Isra Israngkul Na Ayuthaya: Conceptualization, Resources, Investigation, Methodology, Formal analysis, Writing–Original Draft. Taweap Sanghangthum: Methodology, Writing–Review & Editing. Puntiwa Oonsiri, Sornjarod Oonsiri, Mintra Keawsamur, Sirinya Ruangchan, Chulee Vannavijit, Jaruek Kanphet, Sakda Kingkaew, Mananchaya Vimolnoch, Tanawat Tawonwong, and Nuttha Plangpleng: Methodology.

## CONFLICT OF INTEREST STATEMENT

The authors have no relevant conflicts of interest to disclose.
